# Pembrolizumab-induced toxic epidermal necrolysis: case report

**DOI:** 10.1093/omcr/omac025

**Published:** 2022-03-16

**Authors:** Kar Ven Cavan Chow, Connor O’Leary, Fiona Paxton-Hall, Duncan Lambie, Kenneth O’Byrne

**Affiliations:** Medical Oncology, Princess Alexandra Hospital, Woolloongabba, Queensland, Australia; Medical Oncology, Princess Alexandra Hospital, Woolloongabba, Queensland, Australia; Faculty of Health, Queensland University of Technology, Brisbane, Queensland, Australia; Medical Oncology, Princess Alexandra Hospital, Woolloongabba, Queensland, Australia; Anatomical Pathology, Princess Alexandra Hospital, Woolloongabba, Queensland, Australia; Medical Oncology, Princess Alexandra Hospital, Woolloongabba, Queensland, Australia; Faculty of Health, Queensland University of Technology, Brisbane, Queensland, Australia

## Abstract

A 63-year-old man with metastatic lung adenocarcinoma presented with biopsy confirmed toxic epidermal necrolysis (TEN). Symptoms commenced following 3 cycles of carboplatin, pemetrexed and pembrolizumab, with the first cycle given ~9.5 weeks prior to presentation. The patient was managed with immunosuppressive therapy including high dose methylprednisolone, cyclosporine, intravenous immunoglobulin, antibiotics and optimal skin care, and achieved excellent recovery of the skin lesions with minimal sequelae. This rare occurrence of pembrolizumab-induced TEN has only been reported previously in a few cases with limited evidence on management. Given the increasing use of immune checkpoint inhibitors and the long half-life of these agents, our case highlights the importance of recognizing this complication and of a multidisciplinary approach to management.

## INTRODUCTION

Immunotherapy has become an integral component of treatment regimens for various malignancies. Prolonged immune activation can lead to acute, long-term or progressive immune-related adverse effects (irAEs) over time. The overall rates of cutaneous toxicity for programmed death 1 receptor (PD-1), programmed cell death ligand 1 (PD-L1) and cytotoxic T-lymphocyte associated antigen 4 (CTLA-4) inhibitors are 34–42%, 15–20% and 50–70%, respectively. Most reactions are mild, but severe grade 4 reactions, including Steven–Johnson’s Syndrome (SJS) and toxic epidermal necrolysis (TEN) occurs in < 1% of cases [1].

SJS and TEN are rare, life-threatening immune-mediated cutaneous reactions with a mortality rate of up to 30%. TEN is almost exclusively caused by medications whereas SJS can rarely be triggered by infectious agents [2]. SJS/TEN presents with rapidly progressing painful, blistering detachment of the epidermis with the time to maximum epidermal detachment being ~7–10 days. The distinction between both is by the body surface area involvement (BSA), with SJS being < 10%, TEN >  30% and 10–30% being considered as SJS/TEN overlap.

## CASE REPORT

Patient is a 63-year-old man with metastatic lung adenocarcinoma involving the omentum, liver and multiple intracranial lesions. PD-L1 expression was 80% and tests for driver mutations were negative. He completed gamma knife radiosurgery to the brain lesions prior to commencing systemic treatment with pembrolizumab, carboplatin and pemetrexed. Cycle 1 of treatment was given ~9.5 weeks (66 days) before admission and the third cycle was given 17 days earlier. His other medications included perindopril, oxycodone and levetiracetam, the latter commenced post gamma knife for seizure prophylaxis.

**Figure 1:**
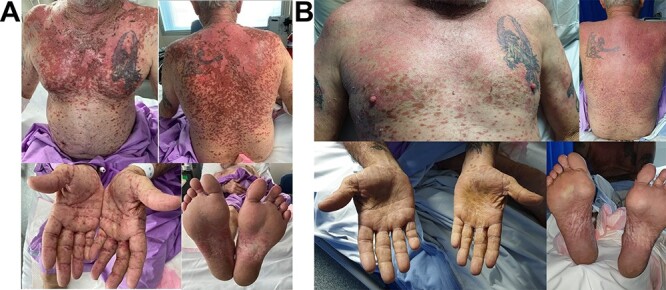
(**A**) Widespread lichenoid eruption, de-epithelialization, blistering and atypical targetoid lesions involving the chest, back, upper limbs, lower limbs, palms and soles. Images taken on admission. (**B**) Re-epithelialization of the skin on affected areas taken prior to discharge.

**Figure 2:**
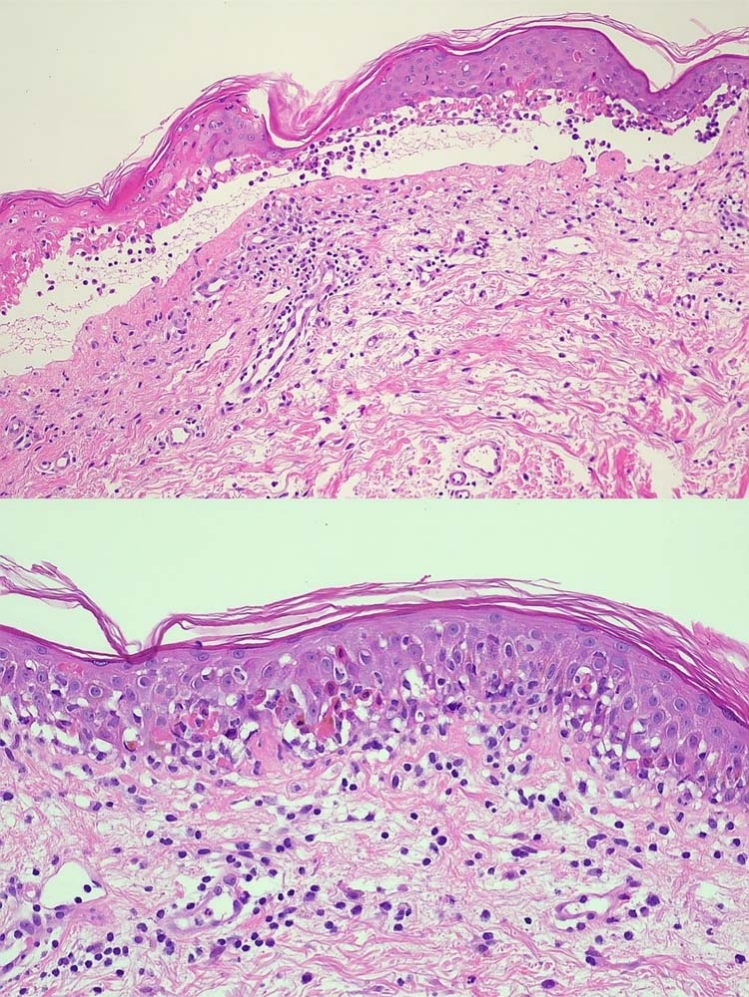
Full thickness epidermal necrosis associated with a subepidermal split. Multiple dyskeratotic cells along the basal layer of the epidermis and at higher levels. Presence of lymphocytes and degenerate keratinocytes with the subepidermal vesicle. Mild superficial perivascular infiltrate of lymphocytes. Findings consistent with TEN.

The patient presented with widespread painful lichenoid eruption with small blisters and atypical targetoid lesions involving the chest, back, limbs, palms and soles (Fig. 1A). Haemorrhagic crusts were seen on the lips with no oral ulcers or ocular involvement. Total BSA involved was >30% with a positive Nikolsky sign. The lesions rapidly progressed over the next few days with epidermal detachment, confluence of the blisters and genital erosion. Histopathology of a skin biopsy showed acute cytotoxic lichenoid reaction characterized by zones of confluent epidermal necrosis, subepidermal vesicle formation and numerous basal and suprabasal apoptotic keratinocytes with some follicular involvement consistent with SJS/TEN (Fig. 2). Tests for potential infectious culprits including varicella-zoster virus, herpes simplex virus, hepatitis B and C were negative.

Levetiracetam was ceased promptly on admission and multiple subspecialties were involved in the patient’s management. He received 7 days of intravenous (IV) methylprednisolone 3 mg/kg daily, IV immunoglobulin (IVIG) 2 g/kg given in divided doses over 3 and 7 days of oral cyclosporin 150 mg BD. Supportive measures with intensive skin care regimens, daily ophthalmology reviews, antihistamines, packed red blood cell transfusions, IV fluids and analgesia were implemented throughout.

The skin lesions progressed and reached a plateau ~1.5 weeks into his admission followed by gradual re-epithelialization. He was subsequently stepped down to oral prednisolone at 1 mg/kg, which was gradually weaned off after 1 month. He achieved excellent recovery with complete skin re-epithelialization and no long-term sequelae (Fig. 1B). He was not rechallenged with any systemic therapy for his non-small cell lung cancer (NSCLC) and was managed as an outpatient with best supportive cares and regular follow ups. The patient passed away 5 months post discharge from disease progression.

## DISCUSSION

We identified three potential culprits in our case—pembrolizumab, pemetrexed and levetiracetam.

The rates of any irAEs are tumour-specific and occur most frequently in the treatment of melanoma and NSCLC, with inconsistent correlation between dose and toxicity across previous studies [3]. There were 18 reported cases of PD-1/PD-L1-induced SJS or TEN in the literature. Of those, pembrolizumab was the causative factor for four cases of SJS and one case of TEN. Amongst all the cases, there were no deaths for the 13 cases of SJS but 3 out of 5 cases of TEN were fatal, including the solitary case of TEN caused by pembrolizumab [3]. The cases were treated with a combination of varying doses of corticosteroids, infliximab, IVIG, cyclosporin and plasmapheresis. The median time of onset for SJS/TEN in pembrolizumab was 11 weeks and the delayed reaction may be explained by the long half-life of 14–27 days which reaches steady state only by approximately Week 18–19 with 3 weekly infusions [3].

Pemetrexed is a multi-targeted antifolate chemotherapeutic agent. Antifolate cytotoxic skin reaction (ACSR) is a known side effect mainly comprizing of mild cutaneous rash occurring shortly after antifolate agent administration [4]. Only six cases of non-fatal SJS/TEN-like skin reactions have been attributed to pemetrexed in the treatment of NSCLC [5–9]. Levetiracetam is a newer generation non-aromatic anti-epileptic drug (AED) often considered as a safer substitute for those who had skin reactions to older AEDs. Cases of SJS/TEN attributed to levetiracetam are extremely rare [10].

The number of SJS/TEN cases due to immune checkpoint inhibitors (ICI) were far greater compared to pemetrexed or levetiracetam, despite the latter two drugs being used for a much longer duration. In our case, TEN occurring after cycle 3 of treatment is also consistent with the average time to onset associated with pembrolizumab. Therefore, we concluded that pembrolizumab is the most likely causative agent.

Management of SJS/TEN is largely supportive, relying on identification and prompt removal of the inciting agent, management in a burns unit, good skin and wound care along with multi-disciplinary consultations [2]. Trials for steroids and immunomodulatory therapies in non-ICI-induced SJS/TEN have shown mixed survival or prognostic benefits [2]. On the contrary, guidelines for skin-related irAEs rely heavily on corticosteroids with addition of other immunomodulatory agents for steroid-refractory cases [1]. Thus, the ideal management and prognosis for ICI-related SJS/TEN remains an uncharted territory.

In conclusion, severe grade 4 skin-related irAEs are rare but important life-threatening conditions which needs prompt recognition and treatment. ICIs have a long half-life which can lead to delayed onset irAEs, therefore a high index of suspicion should be maintained throughout the course of treatment. The ideal immunosuppressive agent, optimal dosing and duration is unclear, and treatment should be tailored towards disease response. Given the severity, we would recommend an aggressive immune suppressive, multi-disciplinary approach as used in other severe ICI toxicities.

## CONFLICT OF INTEREST

No conflict of interest.

## FUNDING

There were no sources of funding.

## CONSENT

Patient’s next-of-kin has reviewed the case content and provided written consent for patient’s case to be published with this journal.

## References

[1] Coleman EL, Olamiju B, Leventhal JS. The life-threatening eruptions of immune checkpoint inhibitor therapy. Clin Dermatol 2020;38:94–104. 10.1016/j.clindermatol.2019.10.015. 32197753

[2] Charlton OA, Harris V, Phan K, Mewton E, Jackson C, Cooper A. Toxic epidermal necrolysis and Steven-Johnson syndrome: a comprehensive review. Adv Wound Care (New Rochelle) 2020;9:426–39. 10.1089/wound.2019.0977. 32520664 PMC7307670

[3] Maloney NJ, Ravi V, Cheng K, Bach DQ, Worswick S. Stevens-Johnson syndrome and toxic epidermal necrolysis-like reactions to checkpoint inhibitors: a systematic review. Int J Dermatol 2020;59:e183–8. 10.1111/ijd.14811. 32052409

[4] Pierard-Franchimont C, Lesuisse M, Humbert P, Delvenne P, E. Pierard G. Toxic epidermal necrolysis and antifolate drugs in cancer chemotherapy. Curr Drug Saf 2012;7:357–60. 10.2174/157488612805076543. 23373549

[5] Huang J-J, Ma S-X, Hou X, Wang Z, Zeng Y-D, Qin T, et al. Toxic epidermal necrolysis related to AP (pemetrexed plus cisplatin) and gefitinib combination therapy in a patient with metastatic non-small cell lung cancer. Chin J Cancer 2015;34:94–8. 10.5732/cjc.014.10151. 25418188 PMC4360078

[6] Bosch-Barrera J, Gaztañaga M, Ceballos J, Pérez-Gracia JL, López-Picazo JM, García-Foncillas J, et al. Toxic epidermal necrolysis related to pemetrexed and carboplatin with vitamin B12 and folic acid supplementation for advanced non-small cell lung cancer. Oncol Res Treat 2009;32:580–4. 10.1159/000232315. 19816075

[7] Eichhoff G . Slowly developing toxic epidermal necrolysis-like reaction associated with pemetrexed and carboplatin. Ecancermedicalscience 2020;14:1010. 10.3332/ecancer.2020.1010. 32256693 PMC7105330

[8] Tummino C, Barlesi F, Tchouhadjian C, Tasei AM, Gaudy-Marqueste C, Richard MA, et al. Severe cutaneous toxicity after pemetrexed as second line treatment for a refractory non small cell lung cancer. Rev Mal Respir 2007;24:635–8. 10.1016/s0761-8425(07)91133-x. 17519817

[9] Scheinpflug K, Menzel C, Koch A, Kahl C, Achenbach HJ. Toxic epidermal necrolysis related to cisplatin and pemetrexed for metastatic non-small cell lung cancer. Oncol Res Treat 2012;35:600–3. 10.1159/000342671. 23038233

[10] Frey N, Bodmer M, Bircher A, Rüegg S, Jick SS, Meier CR, et al. The risk of Stevens-Johnson syndrome and toxic epidermal necrolysis in new users of antiepileptic drugs. Epilepsia 2017;58:2178–85. 10.1111/epi.13925. 29027197

